# Attention-based hybrid CNN-LSTM and spectral data augmentation for COVID-19 diagnosis from cough sound

**DOI:** 10.1007/s10844-022-00707-7

**Published:** 2022-04-23

**Authors:** Skander Hamdi, Mourad Oussalah, Abdelouahab Moussaoui, Mohamed Saidi

**Affiliations:** 1 Department of Computer Science, University of Ferhat Abbes Setif, 19000 Setif, Algeria; 2 Department of Computer Science and Engineering, University of Oulu, 90570 Oulu, Finland

**Keywords:** COVID-19, Cough sound, Convolutional neural network, Long-short term memory, Attention mechanism, Spectral data augmentation

## Abstract

COVID-19 pandemic has fueled the interest in artificial intelligence tools for quick diagnosis to limit virus spreading. Over 60% of people who are infected complain of a dry cough. Cough and other respiratory sounds were used to build diagnosis models in much recent research. We propose in this work, an augmentation pipeline which is applied on the pre-filtered data and uses i) pitch-shifting technique to augment the raw signal and, ii) spectral data augmentation technique SpecAugment to augment the computed mel-spectrograms. A deep learning based architecture that hybridizes convolution neural networks and long-short term memory with an attention mechanism is proposed for building the classification model. The feasibility of the proposed is demonstrated through a set of testing scenarios using the large-scale COUGHVID cough dataset and through a comparison with three baselines models. We have shown that our classification model achieved 91.13% of testing accuracy, 90.93% of sensitivity and an area under the curve of receiver operating characteristic of 91.13%.

## Introduction

COVID-19 is now acknowledged as a new disease from the Severe Acute Respiratory Syndrome CoronaVirus 2 (SARS-CoV2), which is still witnessing a rapid spread in all countries of the world in all its mutated forms. The number of infections until December 15, 2021 reached 270,791,973 confirmed cases with 5,318,216 deaths (Who coronavirus disease (covid-19) dashboard, 2021). The difficulty in controlling the spread of the virus is due to the long incubation period without the appearance of symptoms and the lack of disease diagnosing options. RT-PCR is the golden standard for detecting COVID-19 (Tahamtan & Ardebili, 2020), but the test result can be delayed for several hours, which can question the effectiveness of the patient isolation strategy and subsequent treatment. This is especially stressed in countries with limited RT-PCR test facility resources. On the other hand, it is acknowledged that RT-PCR is not effective for controlling the rapid spread of the virus due to RT-PCR test turnaround time, which may exceed 48 hours in some countries. Likewise, the lack of sufficient quantities of testing kits, possibly for economic reasons, also led to saturation of hospitals and a huge pressure on health management authorities. For these reasons, several studies have been performed on early detection of COVID-19 using alternative cheap solutions, especially using artificial intelligence techniques. For instance, some symptoms of pneumonia diseases such as bacterial pneumonia, pneumonia viral, which bear the same characteristics as COVID-19 pneumonia, can be diagnosed using chest X-ray scans. Many recent works have been published in this respect, deep learning techniques especially CNNs and transfer-learning are widely used (Wang et al., 2020; COVID-19 detection from chest X-Ray images using Deep Learning and Convolutional Neural Networks, 2020; Asif et al., 2020; Berrimi et al., 2021). Similarly, deep learning and machine learning techniques are performed on CT scan images for COVID-19 diagnosis (Berrimi et al., 2021; Singh et al., 2020; Li et al., 2020; Ardakani et al., 2020). Authors in (Ai et al., 2020) showed that CT chest scans achieve a high sensitivity rate of 97% with confidence intervals of 95%, 98% where the ground truth was obtained using RT-PCR tests. Recently, other researchers have explored the analysis of respiratory and coughs sounds for a quick diagnosis of COVID-19 disease. This arises from the observation that a dry cough caused by COVID-19 appears in many COVID-19 patients according to WHO (Who coronavirus disease health topics, 2021), although different from other respiratory coughs. Some researchers have compiled audio databases that include short records of coughs and breathing for COVID-19 positive and negative cases. This motivates the current work in this paper where a novel deep-learning architecture based on a combination of Convolutional Neural Networks (CNNs) and Attention-mechanism based Long-Short Term Memory (LSTM), trained on a large-scale cough dataset called COUGHVID. Our contributions in this paper are the following:

- We provide a concise and a critical summary of related works about COVID-19 diagnosis using cough and respiratory sounds using deep learning techniques.
- We analyze COUGHVID dataset and propose a raw signal and spectral data augmentation, class balancing to create more variability in a way to enhance the efficiency of machine learning and deep learning based solutions.
- We propose a novel framework based on hybrid deep learning attention-based CNN-LSTM architecture that can recognize and distinguish the Likely-COVID-19 from Non-Likely-COVID-19 cases using solely cough sound as input.

This paper is organized as follows: Section 2 details background and related works on COVID-19 early detection from cough. Section 3 highlights the employed dataset and the proposed methodology. Section 4 presents our experimental setup. Sections 5 and 6 present the experimental results and discussions, respectively, where the last section concludes our work.

## Background

Methods like X-Ray and CT scans medical analysis can provide good results in terms of COVID-19 detection accuracy, sometimes even exceeding that of RT-PCR. However, these methods require the physical presence of the patient, which increases the possibility of further spread of infection. This raises the importance of contactless-like analysis. Interests to properties of cough-sound, collected via mobile phone or web portal, and whether this can be utilized to identify COVID-19 have been investigated by some researchers. Coughing is one of the symptoms associated with a large number of chest and respiratory diseases. At the same time, it is also one of the common symptoms of COVID-19 disease, although extra analysis is required to distinguish COVID-19 related coughs from other respiratory diseases.

### Cough and respiratory diseases analysis

There are several studies that have shown that cough has both acoustic and spectral properties. Experiments were conducted to analyze cough before and after the challenge of Methacholine (Thorpe et al., 2001), which is a substance that is inhaled to detect asthma and narrows the airways. The results showed that coughing can provide information that would be useful in diagnosis. In another research study (Chatrzarrin et al., 2011), a comparison between dry and wet coughs has been performed where two spectral features were used. The first one is the number of peaks of the energy envelope, while the second one consists of the power ratio of two frequency bands of second-phase cough signal. The results showed a clear separation between wet and dry coughs. The aforementioned works confirmed that cough has potential to discriminate COVID-19 related coughs from other diseases. However, it is also recognized that the existence of a large number of respiratory and non-respiratory medical conditions that cause coughing (Imran et al., 2020) creates challenges that require special care. Indeed, cough can be rooted back to: hay fever (allergic rhinitis), Inhalation of irritants, Lower respiratory tract infections (bronchitis, pneumonia), Pulmonary embolism, Pneumothorax, Heart failure, Post-nasal drip, Upper respiratory tract infections, Gastro-esophageal reflux, among others (Irwin et al., 2006). Using a cough-sound as the main input, Amrulloh et al. (Amrulloh et al., 2015) built a machine learning model that distinguishes between Pneumonia and Asthma in the pediatric population. The authors used Mel-frequency cepstral coefficients (MFCCs), non-Gaussianity score and Shannon entropy as features trained on a neural network model. The approach achieved 89%, 100%, 89% performance in terms of sensitivity, specificity and Kappa measure, respectively. Pramono et al. (Pramono et al., 2016) proposed a multi-step framework for automatic diagnosis of Pertussis disease. The first step corresponds to the sound event detection where the silent parts were removed to ensure that the audio processing is performed on audio signals. Next, 15 types of features were extracted, which include MFCCs, Zero-crossing Rate, Crest Factor, Energy Level, Spectral Roll-Off, Spectral Kurtosis Coefficient, Spectral Centroid. Finally, a logistic regression was trained on the best nine features, extracted using a sequential feature selection model. Similarly, 12 features were used for whooping sound detection followed by a binary logistic regression classifier to distinguish pertussis and non-pertussis cases. Their approach achieved 92% overall accuracy and 97% positive prediction accuracy in distinguishing Pertussis cases.

### Related works

Several studies investigated the use of deep learning and machine learning methods to quickly diagnose COVID-19 from cough and other respiratory sounds. Lella and Pja (Lella & Pja, 2022) proposed to train a multi-channeled deep convolutional neural network with three levels of features: deep features by removing background noise with Data De-noising Auto Encoder (DAE), Gamma-tone Frequency Cepstral Coefficients (GFCC) filter bank and Improved MFCCs (IMFCCs). The model was trained on the university of Cambridge dataset to recognize five classes: Healthy, COVID-19, Asthma, Pertussis and Bronchitis. For COVID-19 vs. Non-COVID-19, they obtained an accuracy of 95.45% and an F1-score of 96.96% using cough, breath and voice samples modalities. Imran et al. (Imran et al., 2020) developed a mobile application through which an audio recording of a cough is sent to a model represented by a deep CNN architecture that checks the recording if it presents a real / poor / noisy cough signal. If the recording is verified as a cough, it is sent to three other parallel classifiers: Deep Transfer Learning-based Multi Class classifier (DTL-MC), Classical Machine Learning-based Multi Class classifier (CML-MC) and Deep Transfer Learning-based Binary Class classifier (DTL-BC). The system uses Mel-spectrogram as input and a machine learning approach to distinguish four classes: pertussis, bronchitis, COVID-19 and normal. The developed system is shown to achieve an overall accuracy of 92.64% with a sensitivity of 89.14% for the COVID-19 class. In the INTERSPEECH 2021 Computational Paralinguistics Challenge, Schuller et al. (Schuller et al., 2021) used two subsets of University of Cambridge dataset for COVID-19 Cough Sub-challenge to create a challenging baselines of different audio feature extraction techniques and toolkits: ComParE functionals features, BoAW features, deep unsupervised representation learning using the AUDEEP toolkit, and deep feature extraction from pre-trained CNNs using the DEEP SPECTRUM toolkit. CNN, LSTM and Support Vector Machine (SVM) have been used for feature learning and classification. By employing a majority voting of best models, they achieved 73.9% average recall score. Similarly, Brown et al. (Brown et al., 2020) from University of Cambridge proposed a large-scale crowdsourced dataset of respiratory sounds collected using either web or mobile apps. Their results from 6613 users, among which 235 were positive cases of COVID-19, indicated that a fair distinction between COVID-19 and non-COVID-19 users can be achieved using a simple binary machine learning classifier. The employed audio features were initially related to signal duration, onset, tempo, period, RMS Energy, spectral centroid, Roll-off frequency, zero-crossing, 13 first components of MFCCs, Δ-MFCC, Δ^2^;-MFCC. Next, using VGGish, a set of 256 features have been extracted and combined to the result of the first step. Lastly, dimensionality reduction was performed using Principal Component Analysis (PCA). The testing resulted in 80% and 72% for precision and recall, respectively and 82% ROC-AUC. In another work, Mohamed *et al.* (Mohammed et al., 2021) proposed to ensemble a CNN model trained from scratch, VGG16 and Tuned-VGG16 to classify cough sounds as COVID-19 positive or negative cases. The authors collected 20min and 4s for positive class and 4 hours, 30 min and 15 seconds for negative class. To tackle the class imbalance, especially for positive class, and to prevent losing cough features when splitting each audio files, each cough recording has been segmented into non-overlapping coughs. Seven features were employed: mel-spectrum, chroma, tonal spectrogram, power spectrum, MFCC, raw data segment. They achieved 77%, 80% and 71%, for AUC-ROC, precision and recall, respectively. Pahar et al. (Pahar et al., 2021) compared between different machine learning and deep learning techniques: ResNet50 (transfer learning), CNN, LSTM, SVM, logistic regression and multi-layer perceptron (MLP) in the classification of COVID-19 positive and negative cases where the models have been built using Coswara (Krishnan et al., 2020) and Sacros datasets. Synthetic Minority Oversampling TEchnique (SMOTE) has been used for data augmentation and class balancing. MFCCs, MFCCs with appended velocity, MFCCs with appended acceleration, log energies, zero-crossing rate (ZCR) and kurtosis have been used as input features. Transfer learning with ResNet50 achieved 98% AUC and 95% sensitivity. After applying Sequential Forward Selection (SFS) for best feature selection, LSTM achieved the best performance when trained on Coswara and tested on Sarcos: 93.8% AUC and 91% sensitivity. Tena et al. (Tena et al., 2022) proposed a new time-frequency methodology where YAMNet 1 was used to identify cough boundaries among other sound signals. Next, for each cough sample, the signal is turned into a time-frequency representation by using Wigner distribution (WD). Then, a convolution of WD (CWD) was obtained to minimize the interference terms. Recursive Feature Elimination (RFE) was applied to select the most discriminant features among time-frequency features. Their approach achieved an accuracy score of 89.79% using RFE and Random Forest classifier, while the sensitivity score and AUC reached 93.81% and 96.04%, respectively using the same configuration. Harvill et al. (Harvill et al., 2021) proposed to use COUGHVID dataset (Orlandic et al., 2021) in pre-training as an unsupervised learning using Auto regressive Predictive Coding (APC) and DiCOVA challenging dataset (Muguli et al., 2021) for fine-tuning the model. In order to perform Autoregressive Predictive Coding, four LSTM layers were used for unsupervised learning with the aim of minimizing the Mean Squared Error (MSE) to copy the first two layers into the fine-tuning network. The meaning of copying the first two layers is to use the output of the second layer as extracted features in the fine-tuned network. Fine-tuning network is composed of the output of the second layer of APC model followed by 2 Bi-directional LSTM layers. Using the recent spectral augmentation technique SpecAugment (Park et al., 2019) enabled the model to reach 76.81% and 85.35% AUC on validation data and blind (DiCOVA challenging) data (places third out of 29 participants), respectively. Xue et al. (Xue & Salim, 2021) combined Coswara and University of Cambridge datasets and proposed a contrastive pre-training for representation learning from unlabelled data for self-supervised representation. A random masking allowed the Transformer structure (feature encoder) to learn the representations. Then, a downstream phase to fine-tune the feature encoder with labeled data was used. The authors used VGGish, Gated Recurrent Unit (GRU), GRU-CP (CP: Contrastive Pre-training enabled), Transformer, Transformer-CP and ensembling the above different proposed methods. Two mentioned similarity functions have been tested during the downstream phase: *Cosine* and *Bilinear* with different masking rates 0%, 25%, 50%, 75%, and 100%. The best results were achieved by ensembling two Transformer-CPs with different masking rates, yielding 84.43% Accuracy, 73.24% Sensitivity and 90.03% AUC. Similarly, using a deep learning CNN based method, Coppock et al. (Coppock et al., 2021) suggested COVID-19 Identification ResNet (CIdeR) based on ResNet architecture. However, the authors used the concatenation of cough and breathing data. Spectrogram of wav audio files and log spectrograms have been extracted as input features. CIdeR achieved 84.6% AUC. Table 1 summarizes the main published work in this field and exhibits the main constraints and results of each of the aforementioned studies. This partly motivates the current work, which relies on the largest publicly available cough sound dataset -COUGHVID- and attempts to revisit the preprocessing, data augmentation and class balancing pipeline in order to develop an accurate COVID-19 sound based early detection system. A hybrid deep learning Attention-based CNN-LSTM architecture is put forward for this purpose.

**Table 1:** Summary of main published works for COVID-19 diagnosis from cough and other respiratory sounds which used deep learning techniques in their methodologies (feature extraction, cough segmentation, representation learning and classification)

	Data, augmentation and balancing		Results^3^		
Work	Data	Aug^1^/Bal^2^	Acc.	Sensitiv.	AUC
(Lella & Pja, 2022)	Subset of UC	Signal/NU	0.9545	NA	NA
(Imran et al., 2020)	Privately collected	NU/NU	0.9285	0.9457	NA
(Schuller et al., 2021)	Subsets of UC	NU/NU	UAR (0.739)	NA	*NA*
(Brown et al., 2020)	UC	Signal/NU	*NA*	0.81	0.88
(Mohammed et al., 2021)	Collected from Github	Signal/NU	NA	0.71	0.77
(Pahar et al., 2021)	Coswara + Sarcos	Signal/SMOTE	0.9533	0.91	0.938
(Tena et al., 2022)	UC+UL+Coswara+ Pertussis+Virufy	NU/NU	0.8979	0.9381	0.9604
(Harvill et al., 2021)	COUGHVID DiCOVA	Spectrogram/NU	NA	NA	0.7683 0.8535
(Xue & Salim, 2021)	Coswara + UC	NU/NU	0.8443	0.7324	0.9003
(Coppock et al., 2021)	UC	NU/NU	UAR (0.765)	NA	0.846

*NU: not used*, *NA: not available* *UC: University of Cambridge dataset*, *UL: University of Lleida dataset*, *Pertussis:Pertussis dataset*, *UAR: Unweighted Average Recall*, *Acc: Accuracy, Sensitiv: Sensitivity* *Aug/Bal: Augmentation/Balancing*^2^,^3^,^4^

## Materials and Methods

**Fig. 1:**
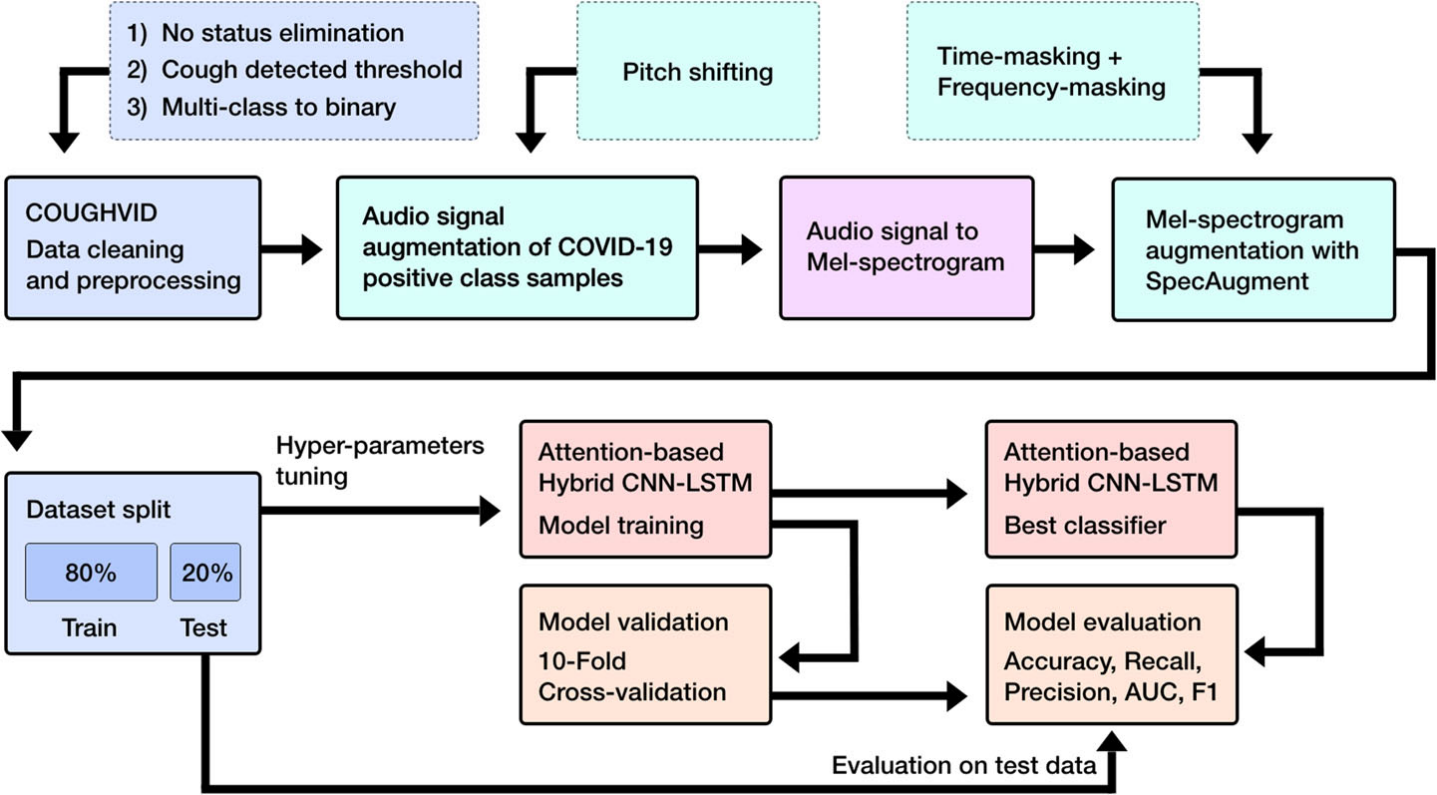
The overall system design of the proposed COVID-19 diagnosis system

Figure 1 presents the overall system architecture of our model for COVID-19 diagnosis from cough sound data. The architecture is composed of several components. First, COUGHVID audio dataset recordings were passed to a pre-processing module. Next, two levels of data augmentation have been applied to both audio signal and spectral data (Mel-spectogram augmentation) to enlarge the training set and deal with the class imbalance problem. The obtained Mel-spectogram features are fed to a new attention-based hybrid CNN-LTSM model to yield binary classification outcomes. The model is validated using a 10-Fold cross-validation where at each epoch, we computed Accuracy, Precision, Recall, F1-score and AUC evaluation metrics to assess the classification performance.

### Dataset

We used COUGHVID crowdsourcing dataset (Orlandic et al., 2021) from École Polytechnique Fédérale de Lausanne (EPFL), Switzerland, which is a large-scale publicly available dataset with 27550 recordings including 1155 positive cases. Recordings were collected through a web application from April 1^*st*^, 2020 to December 1^*st*^, 2020 where users were asked to click on “Record” button to start the recording. After the record process is completed, users were asked to fill in a meta-data questionnaire about *age, gender, geolocation information, previous existing respiratory conditions and COVID-19 status*. The latter includes three status: Healthy, COVID-19 and Symptomatic. Table 2 shows the distribution of COVID-19 status label. The codec of all audio recordings is Opus, with 48kHz sampling rate. For validation purpose, four physician experts assisted in more than 1000 recording annotations. The items that have been annotated were *quality of cough, type of cough, Audible dyspnea, Audible wheezing, Audible stridor, Audible choking, Audible nasal congestion, Nothing specific is audible, impression about the patient’s infection and an impression about the severity*. Interestingly, the meta-data includes an entry called cough_detected *P*_*𝜖*_, which is a float number between 0 and 1 that indicates the extent to which the recording corresponds to a cough or not (probability that the recording is a cough). This value is the output of a machine learning cough classifier and will be discussed in the next section. Other meta-data parameters provided in the dataset description are: *reported_gender, fever_or_muscle_pain, age* and *respiratory_condition*.

**Table 2:** COVID-19 status label distribution over the three statues

	Healthy	Symptomatic	COVID-19	No Status	Total
Samples	12479	2590	1155	11326	27550

### Pre-processing and data cleaning

#### Overall data statistics

We shall notice that COUGHVID dataset includes datums with both known and unknown status. Because we aim to perform a supervised-learning task in this study, we thereby ignored all samples without COVID-19 status (no status provided), which correspond to about 11326 samples. On the other hand, an audio recording may contain *silent* part. For this purpose, we used the Python library Unsilence 5 to remove *silent* parts from all audio recordings in the beginning, the end and the middle (between coughs segments) to keep only the most important vocal patterns. The *silence* removal step reduced the total number of samples to 16082 as there are some empty recordings which were automatically discarded as well. Table 3 shows the new COVID-19 status label distribution after eliminating samples without COVID-19 status and silence removal step.

**Table 3:** Samples count after elimination of *no_status* label with total duration of each class in hours

	Healthy	Symptomatic	COVID-19	Total
Samples	12377 (76.97%)	2567 (15.96%)	1138 (7.07%)	16082 (100%)
Duration [hours]	14.26	3.01	1.44	18.71

#### Cough detection refinement

As pointed out in Section 3.1, under the *cough_detected* entry in the metadata of each record entry is the probability that the corresponding sound is a cough. According to the study carried out in (Orlandic et al., 2021), the authors recommended to use a threshold value of 0.8 as it was found to exhibit the highest precision (95.4%) with eXtreme Gradient Boosting (XGBoost) trained on 68 Prosodic, Spectral and Cepstral features. We want to question this finding by adopting a more prudent attitude, especially with respect to the pre-processing step. We therefore tested the performance of our deep architecture with several potential choice of threshold probability ranging from 0.6 till 0.9. We found the choice of *P*_*𝜖*_ = 0.7 yields the best results. Table 4 shows the new distribution after eliminating all samples which have a *cough_detected* probability threshold under 0.7.

**Table 4:** Samples count and total duration in hours after silence removal and applying *cough_detected = 0.7* threshold

	Healthy	Symptomatic	COVID-19	Total
Samples	8958 (77.06%)	1935 (16.65%)	731 (6.29%)	11624 (100%)
Duration [hours]	9.92	3.10	0.9082	13.92

We therefore employed eXtreme Gradient Boosting (XGBoost) classifier as in (Orlandic et al., 2021) but trained on a randomly selected subset of the original dataset. Besides, we assume that whenever a recording contains both non-cough and cough audio events, it is replaced with a new randomly selected one from the database. We therefore tested the performance of eXtreme classifier, with 68 audio features that include Prosodic, Spectraland Cepstral, on several potential choice of threshold probability ranging from 0.6 till 0.9. We found the choice of *P*_*𝜖*_ = 0.7 yields the best accuracy with a mean AUC of 96.4% and a standard deviation of only 3.3%.

#### Multi-class to binary classification problem

Due to the lack of positive COVID-19 cases (731 compared to 8958), in this study, as reported in our recent work (Hamdi et al., 2021), we combined under **COVID-19**, both the *positive COVID-19* and *Likely-COVID-19* cases, and left the **Non-Likely-COVID-19** (Healthy) unchanged. This turns the multi-class classification scheme into a binary classification scheme. Table 5 shows the new class distribution after merging COVID-19 and Symptomatic classes.

**Table 5:** Samples count after combining COVID-19 and Symptomatic classes

	Non-Likely-COVID-19	Likely-COVID-19	Total
Samples	8958 (77.06%)	2666 (22.94%)	11624 (100%)
Duration [hours]	9.92	4.0	13.92

### Mel-spectrogram

**Fig. 2:**
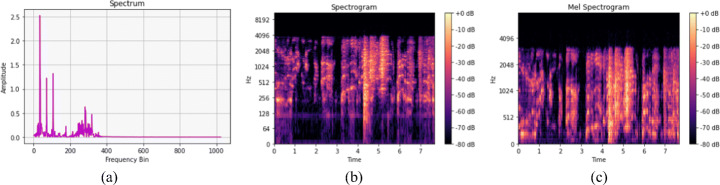
(a) presents a spectrum, (b) shows the spectrogram (STFT and conversion of y-axis to log scale and color dimension to dB) and (c) presents a mel-spectrogram for Likely-COVID-19 case

Spectrograms and Mel-spectrograms (Imran et al., 2020) make use of Fast Fourier Transform (FFT) which performs the Discrete Fourier Transform (DFT) to transform time domain signals *x*(*t*) into frequency domain signals *X*(*f*). Specifically, a particular time domain signal is represented as a sequence of *N* complex integers *x*_0_,...,*x*_*N*− 1_. The FFT of *x*(*t*) is defined by (1)

1 $$ X(f) = {\sum}_{t=1}^{N} x(t) e^{\frac{-2\pi i}{N}(t-1)(k-1)} $$

where, *x*(*t*) represents the sample at time index *t*, *i* is the imaginary number $\sqrt {-1}.X(f)$, and *k* = 0,...,*N* − 1. The result of this transformation, called *Spectrum*, is exemplified in Fig. 2(a). The *Mel-scale*, introduced by Stevens, Volkmann, and Newmann in 1937, is pitch unit that makes identical pitch distances sound similarly far to the listener. It is applied to the frequencies to convert them to the mel-scale (See (2)). Therefore, the *Mel-spectrogram* is the conversion of spectrogram frequencies to the mel-scale as exemplified in Fig. 2(c).

2 $$ mel = 2955 \times log_{10} \frac{1+hertz}{700} $$

### Data augmentation pipeline

We proposed two levels of data augmentation. The first one performs data augmentation on the original audio signal, while the second one focuses on the computed mel-spectrograms. As most of deep learning techniques require a fixed input size, all recordings have been resized to a standard length of 156027 (7.07 seconds) such that samples of more than 7.07 seconds are dropped out while those less than 7.07 seconds are equally padded with zeros at the beginning and at the end. In the next subsections, we explore our two levels of data augmentation.

#### Audio data augmentation with Pitch-Shifting

**Fig. 3:**
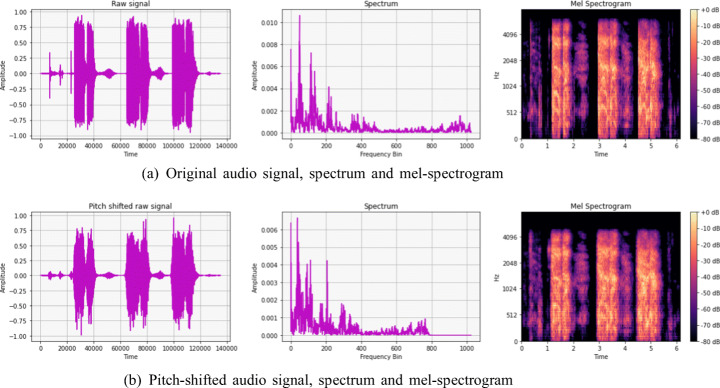
Example of the applied Pitch-shifting for a sample with Non-Likely-COVID-19 class

Because of its simplicity and popularity, we used Pitch Shifting (Lella & Pja, 2022), a sound recording method that raises or lowers the original pitch of a sound. For implementation purpose, we adopted the existing Python library of audio processing and analysis Librosa^6^. This data augmentation is applied only on the *Likely-COVID-19* class in order to increase minority class samples. In overall, audio samples are shifted down by four steps *(n_step = -4)* where a step is defined by a semitone, to generate new samples. The number of steps was chosen after a manual scrutinizing where two independent listeners examined most of the augmented recordings to ensure that vocal features were not affected by pitch shifting. Figure 3 shows the original raw wave, spectrum and mel-spectrogram before and after applying Pitch-shifting method.

#### Spectral data augmentation

**Fig. 4:**
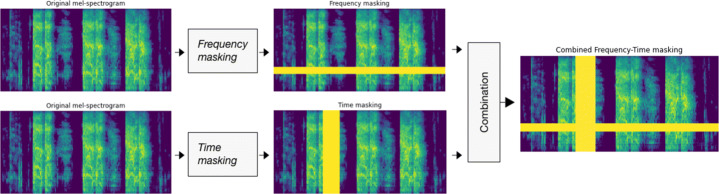
Illustration of SpecAugment, where a new mel-spectrogram is generated by combining *Frequency-masking* and *Time-masking*, for each mel-spectrogram in the dataset

For the purpose of spectral data augmentation and fine-tuning phase, we used *SpecAugment* technique (Park et al., 2019). This is motivated by the finding of DiCOVA challenge where the application of *SpecAugment* led to a substantial improvement of accuracy (see related work Section 2.2). Specifically, *SpecAugment* uses mel-spectrograms and three-step augmentation method. First, using *Time warping*, within the time steps, a random point along the horizontal axis passing through the center of the mel-spectrogram image is warped to the left or right by a distance selected from a uniform distribution ranging from 0 to the time warp parameter along that line. Second, *Frequency masking* is employed as a mechanism of masking *f* consecutive mel frequency channels [*f*_0_, *f*_0_ + *f* ), where *f* is selected from a uniform distribution ranging from 0 to the frequency mask parameter *F*, and where *f*_0_ is selected in [0, *c* — *f* ] (*c* stands for the number of mel frequency channels). The third step consists of *Time masking*, where T successive time steps [*t*_0_, *t*_0_ +*t*) are masked, such that *t* is taken from a uniform distribution from 0 to the time mask parameter *T*, and *t*_0_ is chosen from [0, *τ* — *t*] where *τ* is the number of timesteps of the mel-spectrogram. In our work, we used a combination of *Frequency masking* and *Time masking* with masking parameters *F* = 30 and *T* = 30, respectively, to randomly generate two new mel-spectrograms for the Likely-COVID-19 class in order to solve the class imbalance issue, and one for the Non-Likely-COVID-19 class. Figure 4 shows the process of spectral data augmentation using *SpecAugment*.

### Attention-based hybrid CNN-LSTM

**Fig. 5:**
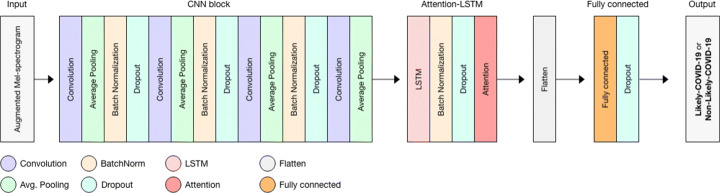
Structure of our proposed Attention Hybrid CNN-LSTM architecture

Our attention-based hybrid CNN-LSTM architecture for COVID-19 diagnosis is shown in Fig. 5. The architecture can be divided into three blocks. The first block uses a CNN architecture, which receives augmented mel-spectrograms as input of shape (39× 88 ×3). Then, the most relevant and informative features are extracted by the convolution layers. In the second block, Attention-LSTM feature maps are passed to LSTM block, where the deep features that have high temporal correlation are selected to be passed to the attention block in order to capture more useful patterns. In the third block, a simple fully connected layer is used for feature learning and classification. Table 6 describes the details of the proposed network architecture in terms of layer type, parameters and output size.

**Table 6:** Details of the proposed network architecture in terms of layer, output shape

#	Layer	Additional information	Output shape
1	Input	Mel-spectrogram of shape (39× 88 ×3)	-
2	Conv2D_1	16 filters of size (2×2), stride (1×1)	(38, 87, 16)
3	AveragePool2D_1	Size (2×2), stride (1×1)	(37, 86, 16)
4	BN_1 + ReLU_1	-	(37, 86, 16)
5	Dropout_1	0.2	(37, 86, 16)
6	Conv2D_2	32 filters of size (2×2), stride (1×1)	(36, 85, 32)
7	AveragePool2D_2	Size (2×2), stride (1×1)	(35, 84, 32)
8	BN_2 + ReLU_2	-	(35, 84, 32)
9	Dropout_2	0.2	(35, 84, 32)
10	Conv2D_3	64 filters of size (2×2), stride (1×1)	(34, 83, 64)
11	AveragePool2D_3	Size (2×2), stride (1×1)	(33, 82, 64)
12	BN_3 + ReLU_3	-	(33, 82, 64)
13	Dropout_3	0.2	(33, 82, 64)
14	Conv2D_4	128 filters of size (2×2), stride (1×1)	(32, 81, 128)
15	AveragePool2D_4	Size (2×2), stride (1×1)	(31, 80, 128)
16	BN_4 + ReLU_4	-	(31, 80, 128)
17	Dropout_4	0.2	(31, 80, 128)
18	Reshape_1	Reshape into recurrent layer input	(31, 10240)
19	LSTM_1	256 units	(31, 256)
20	TanH_5	-	(31, 256)
21	BatchNorm_5	-	(31, 256)
22	Dropout_5	0.2	(31, 256)
23	Attention_1	-	256
24	Flatten_1	-	256
25	Dense_1	100 units	100
26	ReLU_6	-	100
27	Dropout_5	0.5	100
28	Dense_2	1 unit	1
29	Sigmoid_7	-	1

We separated blocks (CNN, LSTM, Attention and Dense) with horizontal lines. *TanH: Hyperbolic Tangent, ReLU* and *Sigmoid* refer to the applied activation functions

#### Convolutional Neural Network (CNN)

We used four convolution layers, with 16, 32, 64 and 128 filters respectively, and a kernel size of (2×2) for each. Each convolution layer is followed by an Average Pooling layer, which is designed to reduce the complexity of the network by linking feature maps to a window with a pre-fixed dimensions, and a stride to define the step unit of the window. In our architecture, we used a pooling window of size (2×2) and a stride of size (1×1). In this block, we used Rectified Linear Unit (ReLU) *f*(*x*) = *max*(0,*x*) as an activation function to increase non-linearity of the feature maps. We used Batch Normalization (BN) to boost the model training by normalizing the activations. In addition, we used Dropout to prevent overfitting and increase model generalization capabilities.

#### Long-Short Term Memory (LSTM)

LSTM is composed of three gates, *input gate, forget gate* and *output gate*. LSTM input gate is given by (3), (4) and (5) where *x*_*t*_ denotes the current input, *h*_*t*_ the current output and *h*_*t*− 1_ the previous output. *C*_*t*_ and *C*_*t*− 1_ refer to the current and previous cell states.

3 $$ i_{t} = \sigma(W_{i} \cdotp [h_{t-1},x_{t}]+b_{i}) $$

4 $$ \widetilde{C}_{t} = tanh(W_{c} \cdotp [h_{t-1},x_{t}]+b_{c}) $$

5 $$ C_{t} = f_{t} * C_{t-1} + i_{t} * \widetilde{C}_{t} $$

Equations 3 and 4 are used to decide which new information is being stored in the cell state by passing *h*_*t*− 1_ and *x*_*t*_ through a sigmoid layer, and through *tanh* layer respectively. *W*_*i*_ and *b*_*i*_ refer to weight matrix and input gate bias, respectively. The new cell state is created by combining the output of sigmoid (3) and *tanh* (4) using (5).

6 $$ f_{t} = \sigma(W_{f} \cdotp [h_{t-1},x_{t}]+b_{f}) $$

Forget gate is denoted by (6), where *W*_*f*_ presents weight matrix and *b*_*f*_ is the offset. Sigmoid and dot product are applied to get a certain probability about forgetting some information from the previous cell.

7 $$ o_{t} = \sigma(W_{o} \cdotp [h_{t-1},x_{t}]+b_{o}) $$

8 $$ h_{t} = o_{t} * tanh({C_{t}}) $$

In (7), *W*_*o*_ and *b*_*o*_ refer to the LSTM’s output gate weight and bias, where *h*_*t*− 1_ and *x*_*t*_ are used to compute the final output, which, in turn, is multiplied by the *tanh* of the state of the new information *C*_*t*_ using (8). In the implementation of this block, the input tensor shape is (31,10240). We used one LSTM layer with 256 units, followed by a Batch Normalization and dropout layers to prevent over-fitting of the network, which is then followed by an attention layer (see next section).

#### Attention mechanism

Attention mechanism focuses the decoder’s attention on the most relevant features of the input sequence using a weighted sum of all previous hidden states. For each time step, given the hidden state of LSTM layer *H*_*t*_ = [*h*_1_,...,*h*_*t*_], *H*_*t*_ is the input of the attention mechanism layer. The attention layer performs three phases: *Scores alignment, Weights* and *Context vector*. In this work, we used the attention mechanism reported in (Xie et al., 2021). We denote the scores alignment by the following equation:

9 $$ S_{t} = tanh(H_{t} \cdot W_{att} + b_{att}) $$

where *W*_*att*_ and *b*_*att*_ are the trainable weights and bias of our attention layer, respectively. The scores *S*_*t*_ are then passed through Softmax function to compute attention weights *α*_*t*_ using the following formula:

10 $$ \alpha_{t} = softmax(S_{t}) $$

After computing attention weights, we then compute the context vector, denoted *attention vector* using (11), which corresponds to a weighted sum of *T* hidden states:

11 $$ a_{t} = {\sum}_{i=1}^{T} \alpha_{t} h_{t} $$

The output of the attention layer is forwarded to a fully connected neural network for feature learning, composed of one layer with 100 units activated by ReLU activation function, followed by a regularization term (Dropout with rate of 0.5).

## Experimental setup

In all experiments, we used *Adamax* algorithm (Kingma & Ba, 2014) as optimizer, which is one of extensions of Adam’s gradient descent algorithm that generalizes the approach to the infinite norm (max) and may result in more effective optimization on particular situations. The output layer of our model architecture has one unit activated by Sigmoid function in order to produce probability *p* of belonging to Non-Likely-COVID-19 (class 0) or Likely-COVID-19 (class 1) (12).

12 $$  y = \begin{cases} 0 & \text{if p $<$ 0.5}\\ 1 & \text{if p $\geq$ 0.5}\\ \end{cases} $$

As loss function, we used Binary Cross Entropy (BCE) which is applied to the scores given by Sigmoid activation function. BCE is formulated by the (13).

13 $$ Loss = -\frac{1}{N} {\sum}_{i=1}^{N} y_{i} \cdot log(\hat{y_{i}}) + (1-y_{i}) \cdot log(1-\hat{y_{i}}) $$

where *N* is the total number of predicted data points, *y*_*i*_ is the real output and $\hat {y_{i}}$ is the predicted output.

### Hyper-parameters tuning

We performed a hyper-parameters tuning stage using the GridSearch for mel-spectrograms computation, data augmentation process, and our classification method in order to find the best hyper-parameters for our deep-learning architecture. We used a sampling rate of 22kHz. We tested different values for *hop_length* (number of samples between consecutive frames), *n_mels* (number of mel frequency bands or the height of spectrogram), *n_ftt* (the size of the FFT computed on the window, before converting to mel bands). For our data augmentation pipeline, we used different values of *n_steps* for Pitch-shifting method and three different values of masking parameter (T and F) while applying SpecAugment. For the classification model architecture, different values of *batch_size*, *learning_rate* have been tested. Table 7 presents the result of this hyper-parameters tuning and selection process.

**Table 7:** Mel-spectrogram computation, data augmentation and classification model architecture hyper-parameters tuning (best value in bold)

	Tested values	**Best value**
hop_length	{128, 256, 512, 1024}	**512**
n_mels	{64, 128, 256}	**128**
n_ftt	{512, 1024, 2048, 4096}	**512**
n_steps	{-1, -2, -3, -4, -5}	**-4**
F (Frequency masking parameter)	{30, 50}	**30**
T (Time masking parameter)	{30, 50}	**30**
batch_size	{64, 128, 256, 512, 1024}	**256**
learning_rate	{1e-05,1e-04,1e-03,1e-02}	**1e-03**

### Training phase

All models including baselines and our proposed model have been trained on Kaggle Notebook 7 which offers 43 hours of GPU usage and 20 hours of TPU per week, 19.6 GB of disk space and 16GB memory, available for 9 hours per session. We used K-Fold cross validation method, which consists of training the classifier on K-1 folds of data and using the rest for validation. This operation is repeated K times for each fold and the final result corresponds to the average of the K experiments. We set *K* = 10. In the first stage, we split the whole data into two subsets, 80% for training and 20% for testing. At each fold, 10% of data is used for validation and the rest for training. As baseline, we trained LSTM, CNN, and hybrid CNN-LSTM without attention mechanism. Each model was trained for 500 epochs.

### Evaluation

We computed six metrics to evaluate the performance of our model and comparison with our baseline models. Components of confusion matrix: True Positive (tp), True Negative (tn), False Positive (fp) and False Negative (fn) have been used to compute the evaluation metrics. *Accuracy* measures the ratio of correct predictions over the total number of evaluated instances. The *Precision* measures the ratio of correct predictions over the number of positive samples. *Recall* or *sensitivity* measures the ratio of true positive predictions over the total number of positive samples. *Specificity* measures the ratio of correctly classified negative cases over the total number of negative samples. *F1-Score* combines recall and precision through harmonic mean. Finally, *AUC* measures the quality of Receiver Operating Characteristic curve (ROC curve which visualizes the tradeoff between true positive rate (TPR) and false positive rate(FPR)). The higher the TPR and the lower FPR, the higher AUC Score. We reported the AUC score for the predicted classes, and probabilities regarding AUC ROC curves.

## Experimental Results

**Fig. 6:**
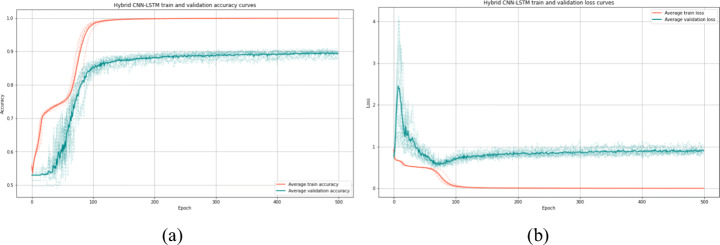
Average accuracy and loss of 10-fold cross-validation for the best baseline model Hybrid CNN-LSTM. (a) for accuracy curves and (b) for loss curves

We computed the aforementioned metrics averaged over training performance after obtaining 10-fold cross-validation results. We note that we used the same set of test in all models, baselines and our proposed model to make a sense for comparison. Results are denoted by average *(avg)* and standard deviation *(std)* of cross-validation *avg ± std*. For LSTM baseline model, we stacked two LSTM layers with 128 and 256 units, respectively. Each layer was followed by a Batch-Normalization layer and dropout (0.2 of rate) followed by a full connection layer with 256 units. Cross-validation took two hours and a half hour. LSTM model achieved an average accuracy of 76.41%, a sensitivity of 73.19% and a ROC AUC score of 76.26%. In the second baseline model (CNN), we used two convolution layers with 16 and 32 filters with the same aforementioned configuration 3.5.1 and a fully connected layer with 64 units. CNN model achieved 83.37% average accuracy, 76.97% sensitivity and 83.00% ROC AUC after 3 hours of training. The last baseline model, hybrid CNN-LSTM, has the same proposed architecture Fig. 5 but without attention mechanism layer. We started with CNN block then passed the feature maps to the LSTM block. It achieved 89.35% average accuracy, 87.74% average sensitivity and 89.28% average ROC AUC. Besides, the training of the third baseline model took 8 hours. Table 8 presents the cross-validation results of the three baselines, Fig. 6 shows the accuracy and loss curves of the best baseline model (Hybrid CNN-LSTM).

**Table 8:** 10-fold cross-validation results of the proposed baselines (LSTM only, CNN only and hybrid CNN-LSTM) in term of the studied evaluation metrics (hybrid CNN-LSTM architecture results are written in bold)

	Accuracy	Sensitivity	Precision	Specificity	F1	AUC-score
LSTM	76.41 ± 0.88	73.19 ± 2.02	75.89 ± 1.41	79.34 ± 0.98	74.48 ± 0.85	76.26 ± 0.87
CNN	83.37 ± 2.07	76.97 ± 4.78	86.31 ± 2.16	89.04 ± 2.47	81.27 ± 2.79	83.00 ± 2.17
CNN-LSTM	89.35 ± 0.76	87.74 ± 1.81	89.46 ± 1.72	90.81 ± 1.42	88.56 ± 0.97	89.28 ± 0.79

**Fig. 7:**
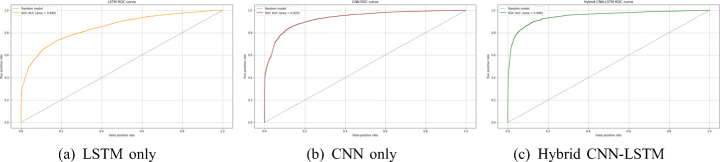
ROC curves of the output probabilities for the three baselines

To validate the obtained results, the performances of the baselines on the test set are shown in Fig. 7 in terms of the ROC curve for output probabilities.

**Fig. 8:**
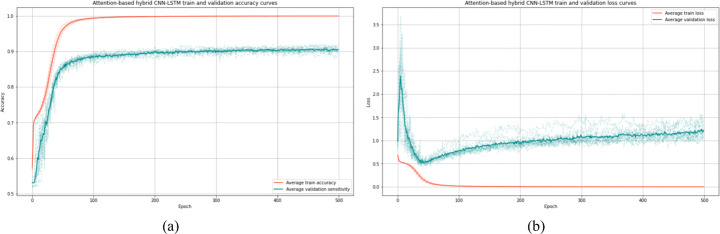
Average accuracy and loss of 10-fold cross-validation for the novel proposed approach Attention-based hybrid CNN-LSTM. (a) for accuracy curves, and (b) for loss curves

We present in Table 9 the details of cross-validation of our approach as well as the overall validation performance after 8 hours of training. In Fig. 8, the accuracy and loss curves are shown to illustrate the training process development of our model.

**Table 9:** 10-fold cross-validation results of the novel approach (Attention-based Hybrid CNN-LSTM) in term of the computed evaluation metrics

	Accuracy	Sensitivity	Precision	Specificity	F1	AUC-score
*avg*±*std*	91.35 ± 0.57	**90.30 ± 0.97**	**91.20 ± 1.19**	**92.27 ± 1.26**	**90.73 ± 0.53**	**91.28 ± 0.54**

**Fig. 9:**
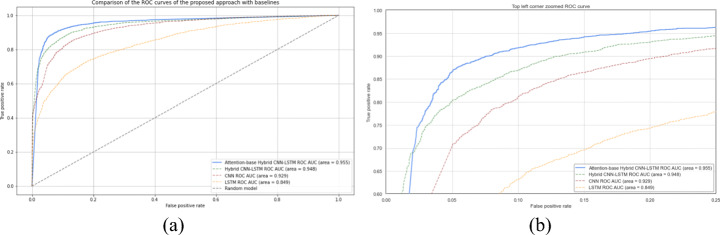
We plot the ROC curve of the baselines compared to our novel approach (a) and we illustrate the differences by zooming the ROC curve on the top left corner (b)

Comparing the proposed approach to baselines reveals in overall an improvement in all evaluation metrics, especially with respect to AUC and sensitivity scores. A full comparison of the baselines with our developed approach is shown in Table 10. We also exhibited the corresponding comparative ROC curves where the superiority of our model is clearly demonstrated (Fig. 9).

**Table 10:** Testing results of the best obtained model on the unseen data for the novel proposed approach compared to the baselines in term of the aforementioned evaluation metrics (best result for each metric is highlighted in bold)

	Accuracy	Sensitivity	Precision	Specificity	F1	AUC-score
LSTM	77.75	72.88	78.76	82.17	75.71	77.52
CNN	85.83	81.53	87.81	89.73	84.55	85.63
CNN-LSTM	88.44	84.41	**90.64**	**92.09**	87.41	88.25
**A-CNN-LSTM**	**91.13**	**90.93**	90.47	91.31	**90.71**	**91.13**

*A-CNN-LSTM* refers to Attention-based Hybrid CNN-LSTM

## Discussion

**Fig. 10:**
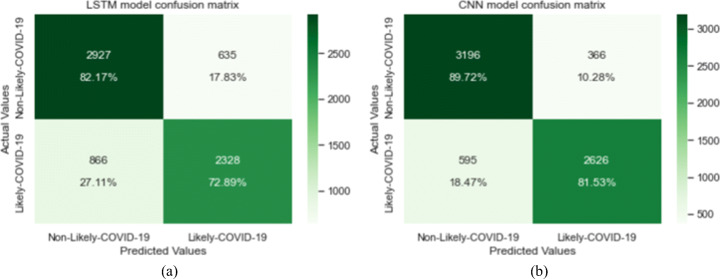
Confusion matrices of the test set. (a) for LSTM model and (b) for CNN model

This work investigated the development of a fast and effective method for diagnosing COVID-19 from solely cough sound recording in order to help limit virus spread. The performance of our framework is demonstrated in the result section of this paper. After pre-processing, filtering original data, we applied the proposed data augmentation pipeline, where we had 8958 negative samples vs. 2666 positive samples, which showed an imbalance class problem. We used the main components of our deep learning based architecture, namely, LSTM, CNN, and LSTM-CNN as baseline models. The results showed that LSTM alone did not work well, with only 77.75% accuracy and 72.88% sensitivity, which led us to conclude that LSTM alone is unable to extract meaningful patterns from mel-spectrogram images. CNN performed better than LSTM in term of correctly classifying Non-Likely-COVID-19 samples (negative), where it achieved 89.73% correct predictions among all negative samples in the test set. We also noted a significant improvement in the sensitivity compared to LSTM, where it was improved from 72.88% to 81.53%. CNN has more ability to extract spatial and spectral features from mel-spectrogram images. Confusion matrices of both models are shown in Fig. 10 where 866 out 3221 positive cases are misclassified and 635 out 3562 negative cases by LSTM classifier (a). Similarly, from the same test set, CNN model (b) misclassified 595 positives samples and 366 negatives cases.

**Fig. 11:**
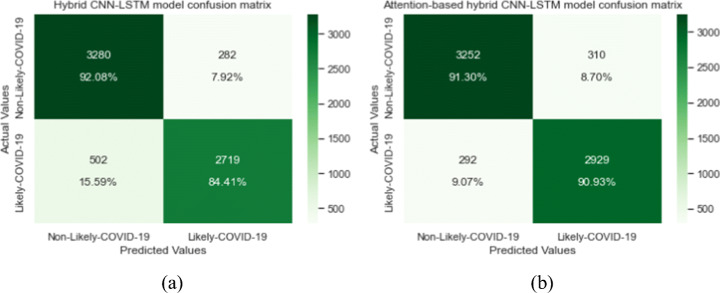
Confusion matrices of the test set. (a) for hybrid CNN-LSTM model and (b) for attention-based hybrid CNN-LSTM model

We thought about hybridizing the aforementioned baselines CNN and LSTM to, first, extract the most important spatial and spectral feature maps using CNN block, and then passing the outcome to LSTM block where temporal correlations between features are extracted. The employed strategy improved and boosted the classification accuracy from 85.83% (achieved by CNN) to 88.44% and a true positive rate of 84.41%. From the confusion matrix in Fig. 11(a), we note the best error rate of 7.92% for negative case where only 282 samples were misclassified, while in case of positive cases, a total of 502 samples were misclassified. We implemented the hybrid model so we can later show the impact of the attention mechanism in the improvement and generalization of our classifier. As we were dealing with a medical case, the need to build a diagnosis system with high performance in terms of sensitivity to reduce possible critical errors (false negatives) is crucial. In the last experiment, we passed the deep extracted temporal features to an attention mechanism module to capture more informative patterns. This proposed approach exhibits the best sensitivity rate of 90.93% compared to the best baseline (hybrid CNN-LSTM with 84.41%) and a classification accuracy of 91.13% and F1-score of 90.71%. In terms of precision and specificity, the best testing results (90.64% and 92.09%, respectively) were achieved by the hybrid CNN-LSTM without attention mechanism. The confusion matrix in Fig. 11 (b) shows that only 292 positive cases were misclassified from 3221 positive testing samples. Our best performing classifier, attention based hybrid CNN-LSTM, was able to distinguish between positive COVID-19 coughs and healthy coughs with an AUC score of 91.13% (95.5% when computing with output probabilities). This comparison is illustrated in Fig. 9(b).

## Conclusion and perspective work

In this study, we developed a COVID-19 diagnosis system from cough sound, we implemented a deep learning based architecture as a classification model. Our model uses COUGHVIG dataset, which is the largest publicly available cough dataset for COVID-19. We have pre-processed the data, then, we applied pitch-shifting on likely positive class samples as the first data augmentation approach. After computing mel-spectrograms, we explored the spectral data augmentation technique SpecAugment for more variety and variability as well as solving the class imbalance issue for positive class (2666 vs. 8958) by combining a randomly applied frequency and time masking on the mel-spectrogram. Two new samples were generated for positive class and one for negative class. We used hybrid CNN-LSTM followed by attention mechanism module while single CNN, LSTM and hybrid CNN-LSTM have been used as baselines to demonstrate the efficiency of our proposal. Our best-performing model is attention-based hybrid CNN-LSTM which achieved 90.93% sensitivity, outperforming the three baselines and an overall classification accuracy of 91.13%. Our model is shown to appropriately discriminate and distinguish between Likely-COVID-19 and Non-Likely-COVID-19 coughs with an AUC score of 0.9113 on the unseen data. We presented a diagnosis system with promising results, fast, easy to deploy and implement. However, our model is also prone to inherent limitations, which can be summarized below:

- **Cough symptom**: One of the most important symptoms of COVID-19 is dry cough. The approach pursued in this paper assumes the cough training data is sufficiently robust to discriminate between COVID-19 and Non-COVID-19 cases. However this assumption can be questioned, since some characteristics of covid-19 cough samples can be found in patients with other diseases as well, e.g., (Tena et al., 2022). However, only healthy versus COVID-19 patients have been considered during the data preparation phase, which ultimately other potential gaps unexplored.
- **Class imbalance**: Although the employed COUGHVID is large-scale dataset with more than 27,000 recordings, the number of positive cases is quite small compared to negative cases (1155 vs 12479), which certainly affects the performance of the proposed system, despite the employed data augmentation strategy.
- **Binary class transformation**: In this study, Positive COVID-19 and Symptomatic classes have been combined to one single class. This process is inevitably accompanied by inherent degradation of the capacity of distinguishing between COVID-19 and common symptoms, which is the reason for our class labeling, Likely-COVID-19 and Non-Likely-COVID-19. However, this may have positive effect of reducing false negative rate. For instance, someone who suffers from dry cough will have more chance to be classified as Likely-COVID-19 and then, more chance to isolate the patient.
- **Model performance and sensitivity**: Our model was able to correctly classify more than 91% of unseen data, and also correctly identified more than 90% of Likely-COVID-19 cases, outperforming some previous studies which worked on different datasets (Brown et al., 2020; Mohammed et al., 2021; Park et al., 2019). However, the error rate for classifying positive cases as negative is 9.07%. This type of error is critical (Type II error) and requires more attention to reduce it further as misclassified patients will not be isolated, which may lead further virus spread.

Furthermore, we believe there is a room for testing and validating the developed approach on alternative cough datasets to demonstrate generalization capability. This will form the basis of our future work.

## Data Availability

All data generated by this work will be made available in Github account of the first author.

## Footnotes

1 https://github.com/tensorflow/models/tree/master/research/audioset/yamnet

2 The modality of augmented data (if used). Signal means that one or more audio data augmentation techniques *(Time-Strechting, Range-Compression, Pitch-Shifting, Background-Noise, Signal-Amplification)* have been applied directly on the raw signal. Spectrogram means that the audio data augmentation is applied after computing the spectrograms.

3 The technique which used for class balancing (if used)

4 As some of works reported the classification of COVID-19 against other respiratory diseases (Pertussis, Asthma and Bronchitis), we only reported results of COVID-19 vs. Healthy tasks

5 https://github.com/lagmoellertim/unsilence

6 https://github.com/librosa/librosa

7 https://kaggle.com/docs/notebooks

## Code Availability

The source code is available in the Github account of the first author. https://github.com/skanderhamdi/attention_cnn_lstm_covid_mel_spectrogram.
